# Metagenomic sequencing combined with flow cytometry facilitated a novel microbial risk assessment framework for bacterial pathogens in municipal wastewater without cultivation

**DOI:** 10.1002/imt2.77

**Published:** 2023-01-05

**Authors:** Songzhe Fu, Rui Wang, Zheng Xu, Huiwen Zhou, Zhiguang Qiu, Lixin Shen, Qian Yang

**Affiliations:** ^1^ Key Laboratory of Environment Controlled Aquaculture (KLECA), Ministry of Education Dalian Ocean University Dalian China; ^2^ Key Laboratory of Resource Biology and Biotechnology in Western China, Ministry of Education Northwest University Xi'an China; ^3^ Shenzhen Yantian District People's Hospital Shenzhen China; ^4^ Institute of Biomedicine and Biotechnology, Shenzhen Institute of Advanced Technology Chinese Academy of Sciences Shenzhen China; ^5^ College of Life Science and Health Northeastern University Shenyang China; ^6^ School of Environment and Energy, Shenzhen Graduate School Peking University Shenzhen China; ^7^ Center for Microbial Ecology and Technology (CMET) Ghent University Gent Belgium

## Abstract

A workflow that combined metagenomic sequencing with flow cytometry was developed. The absolute abundance of pathogens was accurately estimated in mock communities and real samples. Metagenome‐assembled genomes binned from metagenomic data set is robust in phylogenetic analysis and virulence profiling.

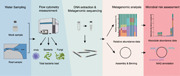

## INTRODUCTION

Microbial assessments of wastewater are essential to control the risks of infection by waterborne diseases [1]. Traditional microbial risk assessment of wastewater is often based on quantification of a few fecal pathogens (e.g., *Escherichia coli* and enterococci), which are often unreliable and time‐consuming [2, 3].

Due to the high sensitivity (as low as 1–10 copies [cp] per ml), qPCR‐based assays have been increasingly employed for the routine surveillance of individual pathogens in clinical and public health settings [4, 5, 6, 7, 8]. However, these assays are constrained by the requirement for a priori knowledge of the pathogens to be targeted [9]. Meanwhile, only a few pathogens can be detected simultaneously in a single test [10]. Thus, early warning of pathogen emergence is limited by the vast diversity and wide range of the pathogen, as well as newly emerging yet‐to‐be‐recognized pathogens.

Recently, environmental metagenomics is becoming an essential technique for pathogen surveillance [11]. Theoretically, this approach could detect all pathogens [12]. In recent years, wastewater surveillance by metagenomics has documented the dynamics of the relative abundance (RA) of bacterial pathogens in municipal wastewater [13, 14], which is emerging as an early‐warning tool for detecting viruses in the local community [15]. Therefore, this approach represents a promising direction for microbial risk assessment of wastewater.

However, due to seasonal shifts in the total bacterial load, RA estimates could misrepresent actual concentrations [16]. These potential deviations may result in the overestimation of microbial risk, as well as the neglect of potential outbreaks. At present, three approaches have been developed for absolute quantification. The first approach is relied on a qPCR assay of a broad‐range 16S rRNA gene to estimate the total bacterial load followed by 16S sequencing to obtain RA data of the bacterial community [16]. Then, the absolute abundance of individual bacterial species can be inferred by multiplying RA data with total bacterial DNA. However, as different organisms contain various gene copy numbers, correcting for 16S rRNA gene copy numbers remains an issue in microbiome surveys [17]. Thus, multiplying RA data by estimates of total bacterial DNA as measured by qPCR assay of a broad‐range 16 S rRNA gene could not provide reliable conclusions. The “spike‐in” of the known absolute abundance of a bacterial species (such as *E. coli*) for calibration is the second approach [18, 19]. However, as the DNA extraction efficiency of different species varies substantially, initial bacterial compositions would be distorted after the DNA extraction [20]. For instance, Urban et al. suggested that the RA value of Enterobacteriaceae is constantly overestimated [13]. Amos et al. also found that the variability of different bioinformatics tools had a significant impact on the estimations of RA [21]. Thus, the spiking method is still inaccurate at the DNA level in reflecting the initial bacterial compositions [22]. The third approach involves the use of direct measurement of the total bacterial load by flow cytometry [23]. Afterward, the absolute abundance of individual pathogens was inferred by multiplying the RA by the total bacterial load. However, this approach was still inaccurate without an RA correction based on the DNA recovery efficiency [24]. Thus, guaranteeing the accuracy of both RA data and the total bacterial load are both essential for approaching the “ground truth” of absolute quantification.

In addition, the inability of the standard microbiome approach to assessing the risks of opportunistic pathogens has been demonstrated [25]. Understanding the virulence profiles of the targeted pathogen is also essential for performing accurate microbial risk assessment, as the infection dose varies remarkably for different virulent types of the same species (etc. O139 or O1 *Vibrio cholerae* strain vs non‐O1/O139 *V. cholerae* strain) [26]. Thus, developing a workflow realizing virulence profiling and absolute quantification of bacterial pathogens simultaneously will provide significant information for microbial risk assessment.

To address these challenges, we first developed a new workflow to infer individual bacterial concentrations in three mock communities by combining metagenomic sequencing with flow cytometry. We revealed that this workflow can provide good estimates of the absolute concentrations of particular bacterial pathogens in both mock and municipal wastewater from a coastal city (Supporting Information: Figure S1), with additional values for obtaining metagenome‐assembled genomes (MAGs) to assess the virulence, antibiotic resistance or functionality of pathogens simultaneously.

## RESULTS

### The rational basis of the workflow

**Figure 1:**
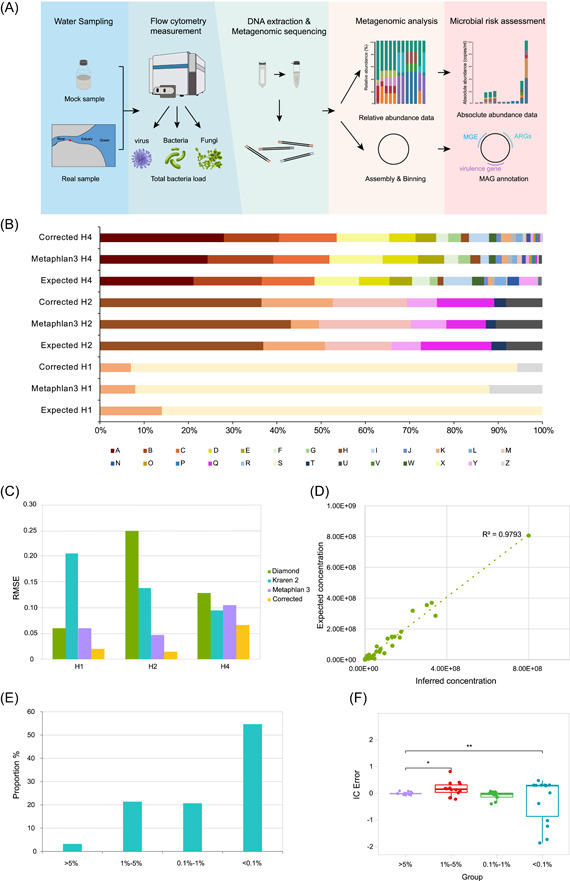
Absolute quantification of individual bacterial species in three mock communities. The workflow of absolute quantification of broad‐range bacterial pathogens and subsequent microbial risk assessment for municipal wastewater (A); MAG, metagenome‐assembled genomes; ARGs, antibiotic resistance genes; Relative abundance of three mock communities estimated by MetaPhlAn3 before and after the correction (B); A, *Acinetobacter johnsonii*; B, *Photobacterium ganghwense*; C, *Acinetobacter pittii*; D, *Aeromonas bestiarum*; E, *Aeromonas caviae*; F, *Aeromonas caviae*; G, *Pseudomonas aestusnigri*; H, *Salmonella enterica*; I, *Bacillus stratosphericus*; J, *Bacillus stratosphericus*; K, *Bacillus cereus*; L, *Shewanella chilikensis*; M, *Vibrio cholerae*; N, *Shewanella xiamenensis*; O, *Vibrio diabolicus*; P, *Klebsiella quasipneumoniae*; Q, *Yersinia pseudotuberculosis*; R, *Citrobacter freundii*; S, *Enterococcus faecalis*; T, *Acinetobacter haemolyticus*; U, *Klebsiella quasipneumoniae*; V, *Vibrio antiquarius*; W, *Shewanella algae*; X, *Acinetobacter junii*; Y, *Klebsiella pneumoniae*; Z, Other; root mean squared errors (RMSE) of three mock communities provided with three classification tools and correction with MetaPhlAn3 (C); Scatter plot of the absolute concentration versus the relative abundance (D); Bar chart of incidence of IC error by relative abundance group (E); Boxplots displaying IC error (Equation 4), with zero inferred concentrations removed, indicate low IC error rates overall (F).

Relative to 16S rRNA amplicon sequencing, we identified two advantages of metagenomic sequencing in our previous study: higher resolution for taxonomy identification and robustness in obtaining MAGs for virulence analysis [25]. In this study, we further employed flow cytometry to quantify the total bacterial load for three mock communities. Subsequently, we obtained the RA values of bacterial species via metagenomic sequencing and converted them into absolute abundance (Figure 1A). Afterward, the number of individual pathogens was inferred. Combined with the virulence profile conferred from MAGs, we were able to justify the microbial risk of certain pathogens in both mock and real water samples.

### Establishment of a method for RA estimation

We first established three mock communities consisting of 2 (H1), 8 (H2), and 32 (H4) bacterial species to assess the best bioinformatic tool for taxonomy profiling (Supporting Information: Table S1) with total assembly sizes ranging from 8.2 to 130 Mb (Supporting Information: Table S2). The RA of bacterial species calculated by MetaPhlan3 was proven to be the most accurate bioinformatic tool for RA estimation (Supporting Information: Figure S2). Afterward, we found that RA was even closer to the actual RA when we considered the variations in the DNA extraction efficiency of individual bacterial species in the three mock communities (Figure 1B) with the lowest RMSE (Figure 1C). Thus, we further evaluated the DNA extraction efficiency of 128 bacterial species from 72 families (Supporting Information: Table S3), which represent the core microbiome in the estuary of this region [25]. The DNA extraction efficiency ranged from 9.6% to 70.8%, with a median value of 41.6% (Supporting Information: Figure S3). The Gram‐negative species also exhibited significantly higher efficiency for DNA recovery (Supporting Information: Figure S3).

### Absolute quantification and binning of individual bacterial species in three mock communities

The total bacterial load was determined using flow cytometry. The values measured by flow cytometry were consistent with the expected values, suggesting the reliability of this approach (Supporting Information: Table S4). In contrast, the use of the qPCR assay based on the universal 16S rRNA gene significantly overestimated the total bacterial load (*p* < 0.05). Next, we calculated inferred concentrations by multiplying the total bacterial load by the corrected RA for each species, as shown in equation 3. The results showed that the inferred bacterial concentration closely tracked the absolute concentration for most species. Linear regression showed a significant correlation between the expected and inferred concentrations (*R* ^2^ = 0.974, *p* < 0.01) (Figure 1D). We defined inferred individual bacteria concentration (IC) error as shown in equation 3. Overall, the mean IC error was low. However, when we further examined the source of IC error, we found that the majority of IC errors tended to originate from bacterial species with lower RA. The results showed that 94.5% of IC errors were identified from the data which RA below 5% (75.5% by an RA below 0.1%), suggesting that the species with low abundance were major sources of IC errors (Figure 1E). The variance in the relationship with expected concentration tended to be inversely proportional to species concentrations (Breusch‒Pagan test; *p* = 0.01) (Figure 1F). Thus, this quantification approach is not suitable for pathogens with RA below 0.1%. MAGs were also obtained in three mock communities to assess the robustness of binning. The N50 values of MAGs ranged from 2317 to 300,286 with a mean value of 91,402, suggesting the good quality of MAGs. In samples H1 and H2, all formulated species were recovered, while in sample H4, 28 MAGs were obtained out of 32 bacterial species. Only four species with RA below 0.13% were not retrieved, indicating the feasibility of binning of pathogen MAGs for microbial risk assessment without cultivation.

### Metagenomic surveillance of bacterial pathogens in the real samples

To evaluate the performance of this new workflow, we tested nine real water samples. From May to September 2021, we conducted semimonthly sampling in the urban wastewaters in Dandong city followed by metagenomic sequencing. Afterward, the RA of individual species was further corrected by normalizing the impacts of DNA extraction efficiency on the ground truth of RA. For those species with unknown DNA extraction efficiency, a median value of 128 bacterial species was applied. We then measured the total bacterial load by flow cytometry. RA estimates were multiplied by the total bacterial load by Equation (3) to obtain the absolute number of individual bacterial species.

Most species showed a consistent trend between RA and IC. However, for some species, such as *Acinetobacter baumannii*, *Acinetobacter pitti*, *Aeromonas caviae*, *Vibrio harveyi*, *Cronobacter sakazakii*, and *Shewanella algae*, the RA changes at some time points were discordant from the IC changes, which often occur when bacterial loads shift dramatically or when the RA is low (Supporting Information: Figure S4). These observations suggested that RA alone is not reliable for indicating the microbial risk level.

Interestingly, relative to the qPCR assay, IC consistently overestimated the absolute concentration by an order of magnitude, most of which were at low RA. Thus, to check whether ICs are predictive of absolute concentrations measured by qPCR, eight pathogens were detected by qPCR for comparison. qPCR results of six pathogens (*A. baumannii*, *Bacillus cereus*, *Salmonella enterica*, *Staphylococcus aureus*, *Vibrio parahaemolyticus*, and *Vibrio cholerae*) revealed that the values obtained from qPCR agreed with the calculated values (*R* ^2^ = 0.1246–0.945, Supporting Information: Figure S5).

**Figure 2:**
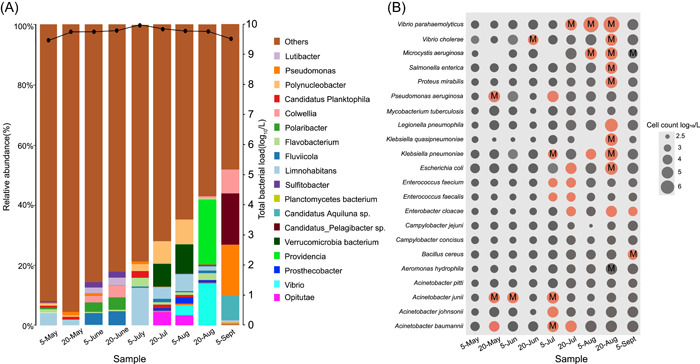
Dynamics of bacterial communities in the municipal wastewater from Dandong city. (A) Relative abundance of bacterial communities at the genus level from May 2021 to September 2021; solid lines are absolute concentrations of total bacterial load measured by flow cytometry. The bar line indicates the relative abundance of bacterial communities at the genus level; (B) Dynamics of bacterial pathogens from May 2021 to Sept 2021; the size of the circle indicates bacterial abundance per ml, which was calculated using the total bacterial load multiplied by the relative abundance of each individual species; Gray: bacterial species detected in the metagenomic data set; Red: bacterial species obtained in pure culture. The letter M in the circles indicates the recovery of the MAGs at specific time points.

The results from flow cytometry showed that the total bacterial load ranged from 7.42 to 9.32 log_10_ cells/L, with a change of 1.9 log_10_ cells/L over 5 months (Figure 2A). A total of 22 potential bacterial pathogens with over 1000 cells/L were abundantly detected in the urban wastewater, with IC values between 2.9 and 6.3 log_10_ cells/L (Figure 2B). The dynamics of inferred individual abundance varied remarkably for each species, of which species belonging to Vibrionaceae and Enterobacteriaceae rose to over 5.0 log_10_ cells/L in July and August.

We further obtained the isolates with high IC. Overall, 21 pathogens were subsequently isolated in pure culture (Figure 2B), of which *A. baumannii*, *V. parahaemolyticus*, *V*. *cholerae*, *S. aureus*, *K. pneumoniae*, *E. coli*, and *E. cloacae* were repeatedly isolated.

## The resolution of MAGs for identification of virulence and antibiotic resistance genes (ARGs)

To test whether metagenomic sequencing with 6G data set was enough for virulence and ARGs profiling, we then binned the contigs obtained from metagenomic datasets. A total of 196 MAGs were recovered. In terms of MAG quality, the mean N50 value, mean completeness, and mean contamination were 23,377, 65.5, and 2.65, respectively (Supporting Information: Table S5). MAGs represented the 78 genera, including 22 pathogens. However, relative to the cultivation, a few pathogen genomes were recovered, such as *Legionella pneumophila*. In addition, MAGs were only recovered with IC over 10^4^ cells/L. For instance, *A. baumannii* was also not recovered in May, although isolates were obtained at that time point.

Moreover, we examined the MAGs for identifying virulence genes and ARGs directly. MAGs and isolates of 14 pathogens obtained at the same time points were selected for comparisons. Binning successfully retrieved all of the key virulence genes and ARGs identified in the above pathogen MAGs (Supporting Information: Table S6).

## DISCUSSION

### Metagenomic sequencing combined with flow cytometry realized the quantification of pathogens of waterborne diseases

In this study, we sought to develop a workflow to evaluate the abundance and virulence profiles of individual bacterial species simultaneously. However, as bioinformatic tools and DNA extraction methods have large impacts on the output of RA [27], in this study, we first established three mock bacterial communities to assess the impacts of DNA extraction and bioinformatic tools on the RA of individual species. The results suggested that the DNA extraction rate is highly variable for different species, ranging from 9.5% to 73.3%. Next, we identify key differences in classification rates and consensus agreements using matched mock community datasets through systematic benchmarking of three different classification tools (MetaPhlAn3, Kraken, and Diamond). Relative to Kraken and Diamond, MetaPhlAn3 has a higher resolution at the species level. After compensation by the DNA extraction rate, the corrected RA from the MetaPhlAn3 outputs showed better agreement with the actual value.

After obtaining the corrected RA, another issue is that RA estimates of individual species often misrepresented actual concentrations due to shifts in total bacterial load. 16S rRNA gene amplicon sequencing combined with flow cytometry has been used as a rapid tool for identifying potential microbial risks [28]. However, as the 16S rRNA gene in bacteria is often found to have multiple copies, previous studies also indicated that 16S rRNA sequencing can only partially detect gut microbiota when compared to shotgun metagenomic sequencing [29]. Thus, we sought to assess the accuracy and boundary of the absolute quantification approach in three mock communities, which suggested that this approach offered a higher resolution than profiling by the universal 16S rRNA gene. Another advantage of our approach is that relative to cultivation, the majority of pathogens with IC over 10^4^ cells/L can be recovered by a new binning tool BASALT developed in our previous study [30]. The limitation of this approach is that as a number of contigs would be lost during the binning, it is possibly hard to distinguish highly similar species within the same genus. Nanopore‐based metagenomic sequencing might overcome this limitation [31]. However, analysis of MAGs from mock communities showed 100% accuracy of taxonomy assignment, indicating this probability is low.

Next, we applied this set of techniques for pathogen surveillance on municipal wastewater from a coastal city. Results of real samples also suggested that the inferred individual bacterial counts showed good agreement with the values obtained by qPCR for six selected pathogens. Meanwhile, virulence genes and ARGs of most pathogen MAGs were recovered. Virulence profiling of MAGs also helps to confirm the virulent type of certain pathogens. For instance, thanks to e identification of *bla*_*KPC‐2*_ and genes encoding hemorrhagic *E. coli* pilus in the *E. coli* MAGs recovered, accurate microbial risk assessment can be accomplished. Based on the abundance and 50% infection dose (ID_50_) of this virulent type [32], we can define that there was a high risk of *E. coli‐related* infection in August.

## CONCLUSION

In this study, we developed a workflow combining metagenomic sequencing and flow cytometry and evaluated its robustness for absolute quantification and microbial risk assessment of specific pathogens in mock and real samples. Results showed that this workflow accurately estimated the absolute abundance of pathogens in mock communities and real samples. Genomic information extracted from MAGs has further led to microbial risk assessment of bacterial virulence and ARG profiles and the identification of novel virulent strains in challenging settings.

## METHODS

### Determination of DNA extraction efficiency from 128 bacterial species

A total of 128 bacterial cultures were obtained from the China General Microbiological Culture Collection Center or previous studies [25]. These 128 species span 94 genera and 72 families (Supporting Information: Table S2), which accounted for over 80% of the regional core estuary bacterial communities locally [25]. The media and cultivation conditions used for liquid cultures were shown in Supporting Information: Table S2 as described in a previous study [25]. Acridine orange direct count was used to determine the cell count.

The bacterial species (500 ml) from sterilized synthetic wastewater were filtered individually through the mentioned GTTP membranes (Merck Millipore). Genomic DNA extracted from the mixed cultures was described previously by Boström et al. [33]. After measuring the total DNA concentrations by Quant‐iT PicoGreen dsDNA Assay Kit (Invitrogen), genome copy numbers were converted based on the genomes' known molecular weight (described by Tourlousse et al.) [27].

The measurement of DNA extraction efficiency was calculated by the following Equation (1):

(1) DNA extraction efficiency(%)=genome copy numbers/cell counts,

where cell counts are the abundance of specific species before DNA extraction.

The corrected relative abundance (CRA) of individual species was inferred by the following Equation (2):

(2) $$\mathrm{CRA}\left(i\right)=\frac{\mathrm{RA}\left(i\right)/\mathrm{DR}(i)}{\sum \left(\frac{\mathrm{RA}\left(1\right)}{\mathrm{DR}\left(1\right)}+\frac{\mathrm{RA}\left(2\right)}{\mathrm{DR}\left(2\right)}+\text{⋯}+\frac{\mathrm{RA}\left(n\right)}{\mathrm{DR}\left(n\right)}\right)},$$

where RA(*i*) is the relative abundance of species *i*, DR is the determined DNA extraction rate for species *i*, CRA(*i*) is the corrected relative abundance for species *i* and RA (1) to RA (*n*) indicate the relative abundance of species 1 to species *n*.

The flow cytometry following the staining of cells was used for cell counts through SYTO 9 green fluorescent nucleic acid stain, which was performed by a CytoFLEX (Beckman Coulter) flow cytometer equipped with a 488‐nm laser [28]. After obtaining the total bacterial load by flow cytometry, the inferred individual bacterial concentration (IC) was calculated by the following equation:

(3) IC(i)=total bacteria lload ∗ CRA(i),

where IC (*i*) is the inferred individual bacterial concentration of species *i*. The data are presented on a log_10_ scale. To keep all values finite when working with a log_10_ scale, zero inferred concentrations were mapped to 0.

### Preparation, metagenomic sequencing, and binning of the bacterial mock community

To assess the influences on the RA of a given pathogen by the DNA extraction and bioinformatics tools in the metagenomic data set, 2, 8, and 32 bacterial species were selected from above 128 bacterial species to construct the three mock communities (namely, H1, H2, and H4) (Supporting Information: Table S1).

The media and cultivation conditions used for the overnight cultures of the above species were shown in Supporting Information: Table S1. The total cell counts of species were measured individually by acridine orange direct count [29]. After pooling 2, 8, and 32 strains together, respectively, each 100 μl of monoculture was transferred into a tube to finally make three mock communities. Followed by the NovoSeq Nano DNA Sample Preparation Guide, DNA samples were prepared accordingly. Sequencing was performed by the platform of an Illumina NovoSeq sequencer (Illumina Inc.) at Novogenes (Tianjin).

Raw reads from Illumina metagenomic sequencing were trimmed and filtered by FastX Toolkit to remove the low quality (*Q*  ≤  20) and short reads (length ≤ 45). The clean reads were then analyzed on the online platform BMK Cloud (www.biocloud.net). Subsequently, the high‐quality clean reads were assembled by SOAPDenovo v1.06 to obtain the scaftigs (minimum length above 500 bp) [34]. Taxonomic profiling of three mock communities was performed by MetaPhlAn3 (v3.0.13) [35], Kraken2 (v 2.1.1) [36], and Diamond (v1.1) [37], respectively (Details see supplementary text).

To obtain MAGs, filtered reads from both mock community and wastewater samples were assembled using SPAdes version 3.2 [38] specifying k‐mer size values at 21, 33, 55, and 77, and finally reserved assembled contigs at lengths of ≥1,000 bps. Contigs were binned into MAGs (completeness ≥ 50% and contamination ≤ 10%.) and processed with BASALT [30].

### Evaluation of the accuracy and boundary of inferred concentrations

To assess the accuracy and boundary of inferred concentrations, the error of IC (IC error) was defined based on Equation (4) to assess the accuracy:

(4) ICerror=log(EC)−log(IC),

where EC is the expected concentration of individual species, IC is the inferred concentration of individual species. The data were converted to log10 values for relative abundance and inferred as expected concentrations.

### Validation of absolute quantification workflow for real samples

The municipal wastewater samples were collected from May to September 2021 in Dandong city (Supporting Information: Figure S1). On each sampling day, 3 L of wastewater was collected at 9:00 a.m. and 3:00 p.m. before pooling together for DNA extraction. All samples were placed in sterile containers immediately and transported to the laboratory via ice package within 8 h. The DNA of wastewater samples was extracted and sequenced as described previously. To assess whether there was any discordance between RA and IC, a qPCR assay was performed for six species, including *A. baumannii*, *A. pitti*, *A. caviae*, *V. harveyi*, *C. sakazakii*, and *S. algae*, as described previously [25]. qPCR was also conducted to quantify another five pathogenic species (*B. cereus*, *S. enterica*, *S. aureus*, *V. parahaemolyticus*, and *V*. *cholerae*) (DAAN Gene, Guangzhou, China) to check the linear relationship between IC and expected abundance.

### Isolation and whole genome sequencing of bacterial pathogens from wastewater

The bacterial pathogens were then isolated from the wastewater samples. In light of the National Food Safety Standard of the People's Republic of China (GB4789), Foodborne pathogens were isolated and characterized as described previously [25]. After overnight bacterial culturing on trypticase soy agar, genomic DNA was extracted and sequenced on the Illumina HiSeq. 2500 platform at Beijing Novogene. Raw reads from the chromosomes were de novo assembled by SPAdes version 3.2 [38]. The virulence factors of MAGs and isolated genomes were identified using the Virulence Factors of Pathogenic Bacteria (VFDB) database [39]. ARGs were identified using ResFinder [40].

### Statistical analysis

The data from qPCR and flow cytometry (mean ± standard deviation, *n* = 3) were compared with one‐way analysis of variance, followed by Tukey's post‐hoc test in R package (R 3.4.1) ggpubr and visualized using ggplot2 [41]. Additionally, Breusch‒Pagan test was performed to confirm the heteroscedasticity of errors in linear regression. Differences among samples were recognized to be statistically significant at *p* < 0.05.

## AUTHOR CONTRIBUTIONS

**Songzhe Fu**: Conceptualization, methodology, writing – original draft. **Zheng Xu**: Software, data curation, writing – review and editing, and visualization. **Rui Wang**: Methodology, validation, software, investigation, and data curation. **Huiwen Zhou**: Validation, software, and investigation. **Zhiguang Qiu**: Validation, software, investigation, and data curation. **Lixin Shen**: Writing – review and editing, and funding acquisition. **Qian Yang**: Conceptualization, supervision, and writing – review, and editing. All authors have approved the final version of the manuscript.

## CONFLICT OF INTEREST

The authors declare no conflict of interest.

## Supporting information

Supporting information.

Supporting information.

## Data Availability

The raw sequencing data were submitted to GenBank (NCBI) under BioProject No. PRJNA860773. Supplementary materials (figures, tables, and graphical abstract) can be found in iMeta Science http://www.imeta.science/.
