# Supplementary material for: Metagenomic sequencing combined with flow cytometry facilitated a novel microbial risk assessment framework for bacterial pathogens in municipal wastewater without cultivation
Source: Imeta. 2023 Jan 5;2(1):e77. doi: 10.1002/imt2.77 (PMC10989823; doi:10.1002/imt2.77)
Supplement: Supplementary file 2 — Supporting information. [file IMT2-2-e77-s001.docx]

**Supplementary material**

**Metagenomic sequencing combined with flow cytometry facilitated a novel microbial risk assessment framework for bacterial pathogens in municipal wastewater without cultivation**

Songzhe Fu^1,6#^, Rui Wang^1#^, Zheng Xu^2,3#^, Huiwen Zhou^4^, Zhiguang Qiu^5^, Lixin Shen^6^, Qian Yang^7^

1. Key Laboratory of Environment Controlled Aquaculture (KLECA), Ministry of Education, Dalian Ocean University, 116023, Dalian, China;
2. Shenzhen Yantian District People's Hospital，Shenzhen 518081, Guangdong, China
3. Institute of Biomedicine and Biotechnology, Shenzhen Institute of Advanced Technology, Chinese Academy of Sciences, Shenzhen 518055, Guangdong, China
4. College of Life Science and Health, Northeastern University, Shenyang, China
5. School of Environment and Energy, Shenzhen Graduate School, Peking University, Shenzhen, 518055, China
6. Key Laboratory of Resource Biology and Biotechnology in Western China, Ministry of Education, Northwest University, Xi'an, 710069, China
7. Center for Microbial Ecology and Technology (CMET), Ghent University, Coupure Links 653, 9000 Gent, Belgium

# Contributing equally to this paper

Author for correspondence: Songzhe Fu

Email: fusongzhe@126.com

Qian Yang

Email: [qian.yang@ugent.be](mailto:qian.yang@ugent.be)

**Supplementary text**

**The impacts of the bioinformatics tool on the relative abundance (RA) of three mock bacterial communities**

We first established three mock bacterial communities spiked with two (sample H1), eight (H2), and 32 species (H4). DNA samples were prepared following the NovoSeq Nano DNA Sample Preparation Guide. The fragmented genomic DNA was used for library construction with the Illumina Nextera® XT Library Prep kit (Illumina, USA) with insertion size of 350 bp. The library was purified with Agencourt AMPure XP beads. Sequencing was then performed using an Illumina NovoSeq sequencer (Illumina Inc., San Diego, CA, USA) with the paired-end 2×150 bp protocol at sequencing depth of 20X and at Novogenes (Tianjin). The raw sequencing data were submitted to GenBank (NCBI) under BioProject No. PRJNA860773.

The raw meta-genomic reads were processed using Fastp (v0.21.0, default settings) to remove adapters and low-quality reads. Three common bioinformatics tools, MetaPhlAn3 (v3.0.13), Kraken2 (v 2.1.1), and Diamond (v1.1), were used for taxonomic profiling in three mock communities. MetaPhlAn3 uses clade-specific marker genes to profile the composition of microbial communities, while Kraken2 calculates the microbial taxon in community samples by a Kmer-based classification algorithm. MetaPhlAn3 was applied against the MetaPhlAn database and minikraken database (8GB, v20200312), respectively. Taxonomic classification was also conducted on the contigs using Kraken2 version 2.1.1 with default parameters and an in-house database containing all Complete Genome from NCBI RefSeq Genome entries (database accessed on October 15, 2021). For the Diamond approach, DNA sequences were mapped into the gene catalog in NR databases using DIAMOND with default parameters (e value ≤1 e-5), and the relative abundance of genes was summed at the same taxonomy level as the relative abundance of each species.

The actual values of each taxon were measured and mixed in a given proportion (Table S2). Metagenomic sequencing was conducted for three mock bacterial communities. The raw meta-genomic reads were processed using Fastp (v0.21.0, default settings) to remove adapters and low-quality reads. Three common bioinformatics tools, MetaPhlAn3 (v3.0.13), Kraken2 (v 2.1.1), and Diamond (v1.1), were used for taxonomic profiling in three mock communities. MetaPhlAn3 uses clade-specific marker genes to profile the composition of microbial communities, while Kraken2 calculates the microbial taxon in community samples by a Kmer-based classification algorithm. These two computational tools were applied against the MetaPhlAn database and minikraken database (8GB, v20200312), respectively. For the Diamond approach, DNA sequences were mapped into the gene catalog in NR databases using DIAMOND with default parameters (e value ≤1 e-5), and the relative abundance of genes was summed at the same taxonomy level as the relative abundance of each species. Bioinformatic tools were tested following the developer’s recommended settings to ensure a fair comparison among these tools. Next, we assessed the number of unclassified reads and misclassified reads (i.e., sequences not assigned to any of the seven bacterial families). The results obtained from the three approaches were compared with the actual values; the deviation between the observed and expected values was measured by the root mean squared error (RMSE).

The results showed that compared with true RA, all three tools disproportionally estimated RA. MetaPhlAn3 was the only bioinformatics tool covering all spiked species in three mock communities, with multiple tools missing the targeted species and detecting a high number of incorrect species at low abundances. Our results indicate that very fast implementations such as Kraken2 yielded less accurate classifications than MetaPhlAn3. Using MetaPhlAn3, 96.2% of sequencing reads were assigned to a bacterial species on average, thereby primarily encouraging automated analyses on the genus (95.6% assigned) or family level (99.6% assigned).

In the two-component sample (H1), the proportions of *E. faecalis* and *B. cereus* were 86.00% and 14.00%, respectively (Figure 1B). Among the three tools, Metaphlan3 and Diamond performed with a higher classification rate of sequence reads into correct bacterial species (88.00%) than Kraken2 (70.52%). Specifically, Metaphlan3 and Diamond performed closer to the expected proportion of *E. faecalis* at 80.00%, while the proportion of *B. cereus* calculated closer to the expected value by Kraken2 was 13.62%. In samples H2 and H4, Metaphlan3 performed with the highest classification rate into correct bacterial species (97.44%) compared to Kraken2 (63.34%) and Diamond (34.30%) (Figure 1C). In sample H2, the read proportions of *Salmonella enterica*, *Vibrio cholerae* and *Klebsiella quasipneumoniae* were 41.15%, 19.81% and 10.05%, respectively, using Metaphlan3, which were slightly higher than expected at 34.78%, 14.17% and 7.70%, respectively. On the other hand, the read proportions of *Yersinia pseudotuberculosis*, *Bacillus cereus* and *Enterococcus faecalis* were 8.51%, 6.04% and 2.09%, respectively, using Metaphlan3, which were lower than expected at 15.00%, 13.13% and 5.83%, respectively. The classification rate of correct bacterial species by Kraken2 was better than that by Diamond, indicating that the read proportion of target bacterial species was universally lower than expected. Notably, sequencing reads could not be classified into *Acinetobacter hemolyticus* by Kraken2 (0.00%) compared to the expected proportion at 3.17%. In the more complex sample H4, Metaphlan3 was still found to have the best classification rate of 99.99% compared to Kraken2 (50.50%) and Diamond (29.47%). Similar to sample H2, Metaphlan3 acquired a slightly higher rate of sequencing reads than expected on bacterial species with higher abundances, such as *Acinetobacter johnsonii*, *Photobacterium ganghwense* and *Acinetobacter pittii* at 23.02%, 14.03% and 11.99% compared to 18.21%, 13.28% and 10.23%, respectively. In contrast, Kraken2 and Diamond exhibited poorer classification rates in more complex samples.

**Normalization of the RA of three mock bacterial communities measured by MetaPhlAn3**

Although the above results clearly showed that overall, the RAs measured by MetaPhlAn3 agreed well with the expected values, some discrepancies were still observed in some genera, especially for *Bacillus* spp.. These results might be associated with the distinct DNA extraction recovery rate among species, especially for Gram-positive species.

Next, we performed DNA extraction for 40 bacterial species and evaluated the recovery rate over different species. Overall, investigated bacterial species had a distinct recovery rate, ranging from 9.64% to 74.8%. In contrast, gram-negative species had a much higher recovery rate, with the highest value (74.8%) for *Klebsiella quasipneumoniae*. Furthermore, we normalized the RA of three mock bacterial communities by Eq. (1) to compensate for the variations in the DNA extraction recovery rate on the actual RA (Figure S2). The results showed that most of the corrected RAs of individual species were closer to the expected RAs. Accordingly, the RMSE for corrected RA is significantly lower than raw RA outputs from MetaPhlAn3. Comparable and high agreement with the ground truth was observed after the correction, suggesting that this method might improve the RA estimation.

**Supplementary Figure**

**Figure S1** The sampling sites in Dandong city were mapped by the ArcGIS Desktop 10.2 software (<http://desktop.arcgis.com/>). CHN: China; DPRK: Democratic People's Republic of Korea


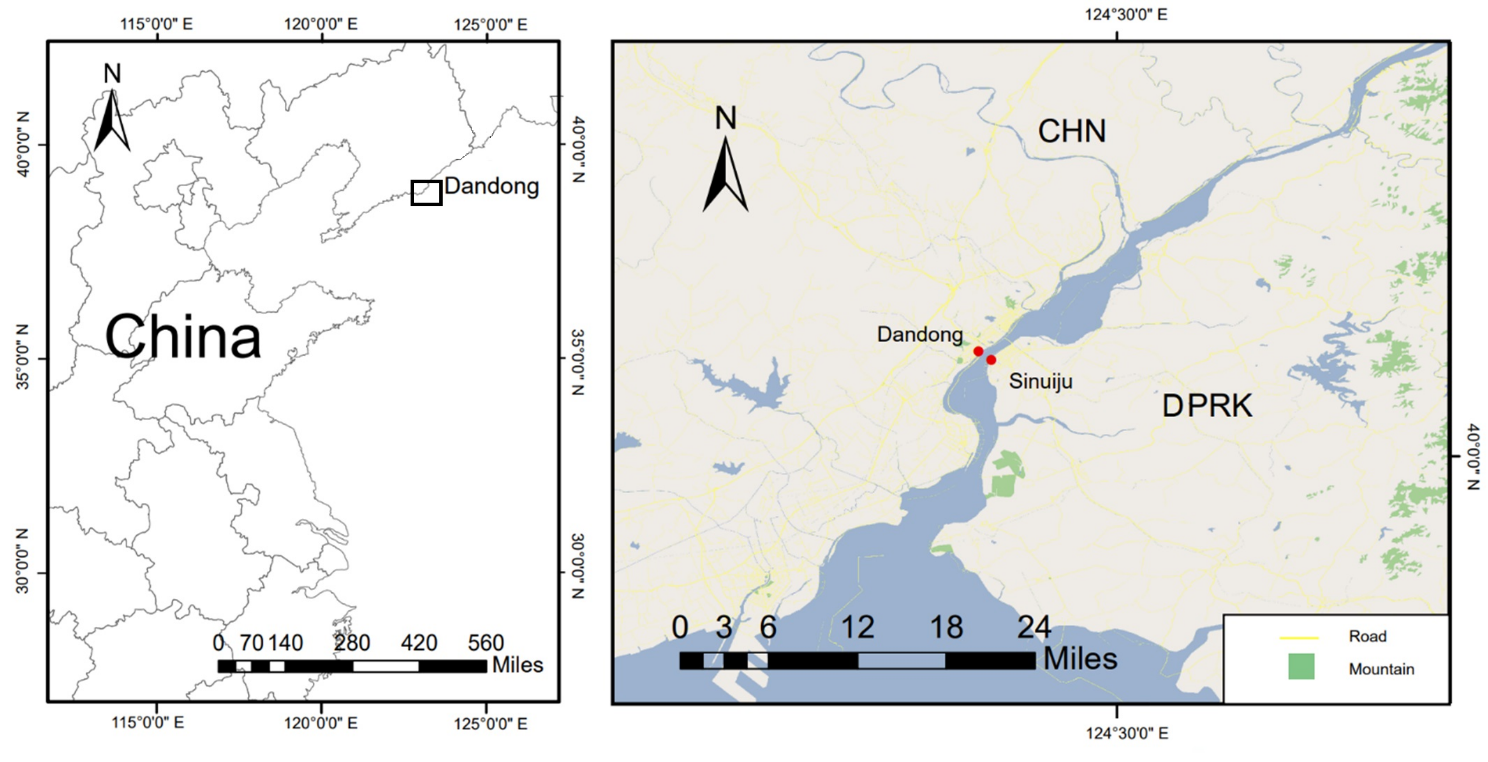


**Figure S2** Benchmarking of three classification tools with metagenomic sequences for three mock communities. A: Relative abundance (RA) of mock community H1 with 2 species estimated by three classification tools; B: RA of mock community H2 with 8 species estimated by three classification tools; C: RA of mock community H4 with 32 species estimated by three classification tools.

**
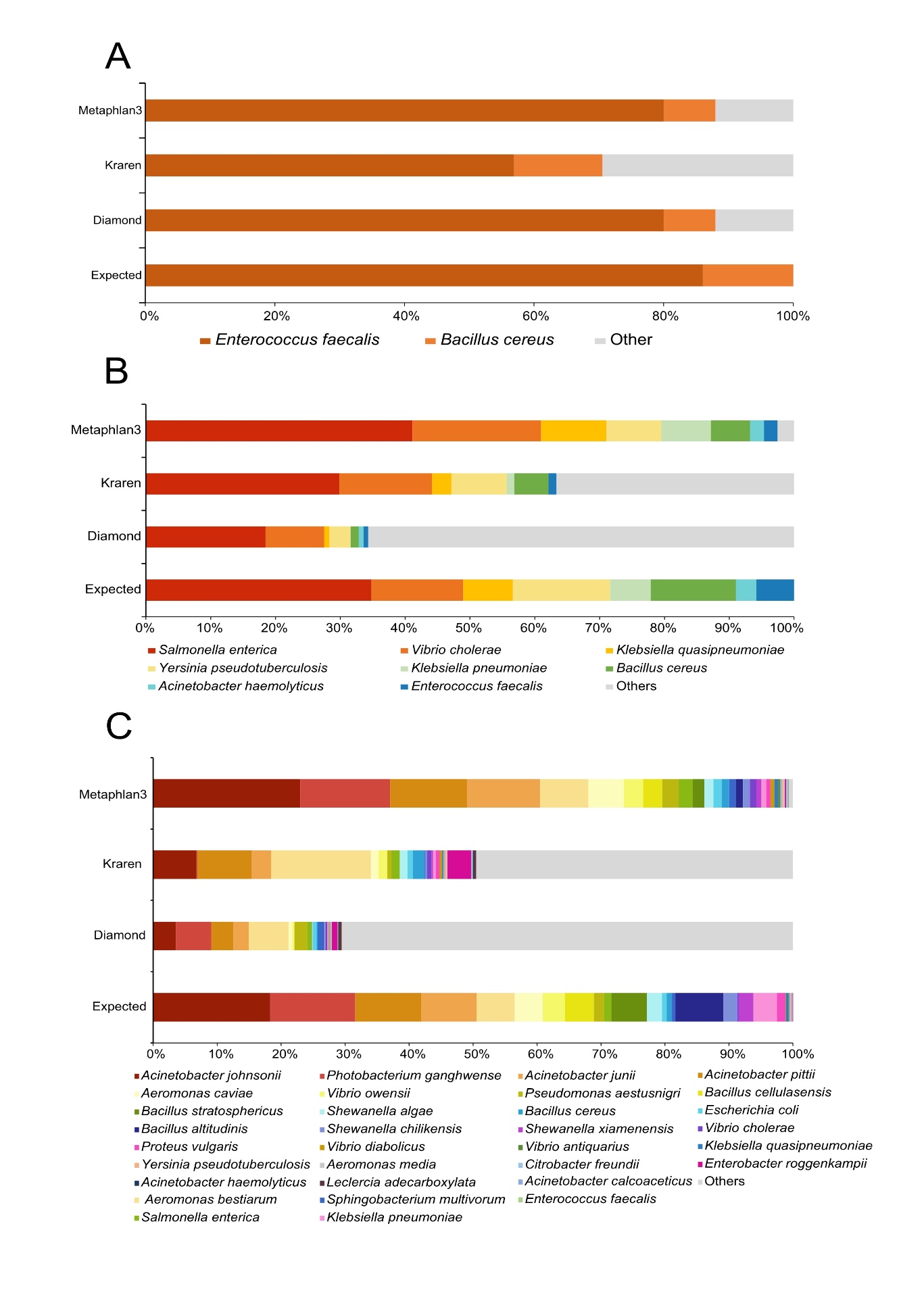
**

**Figure S3** The boxplot of DNA extraction rate of 72 bacterial families.


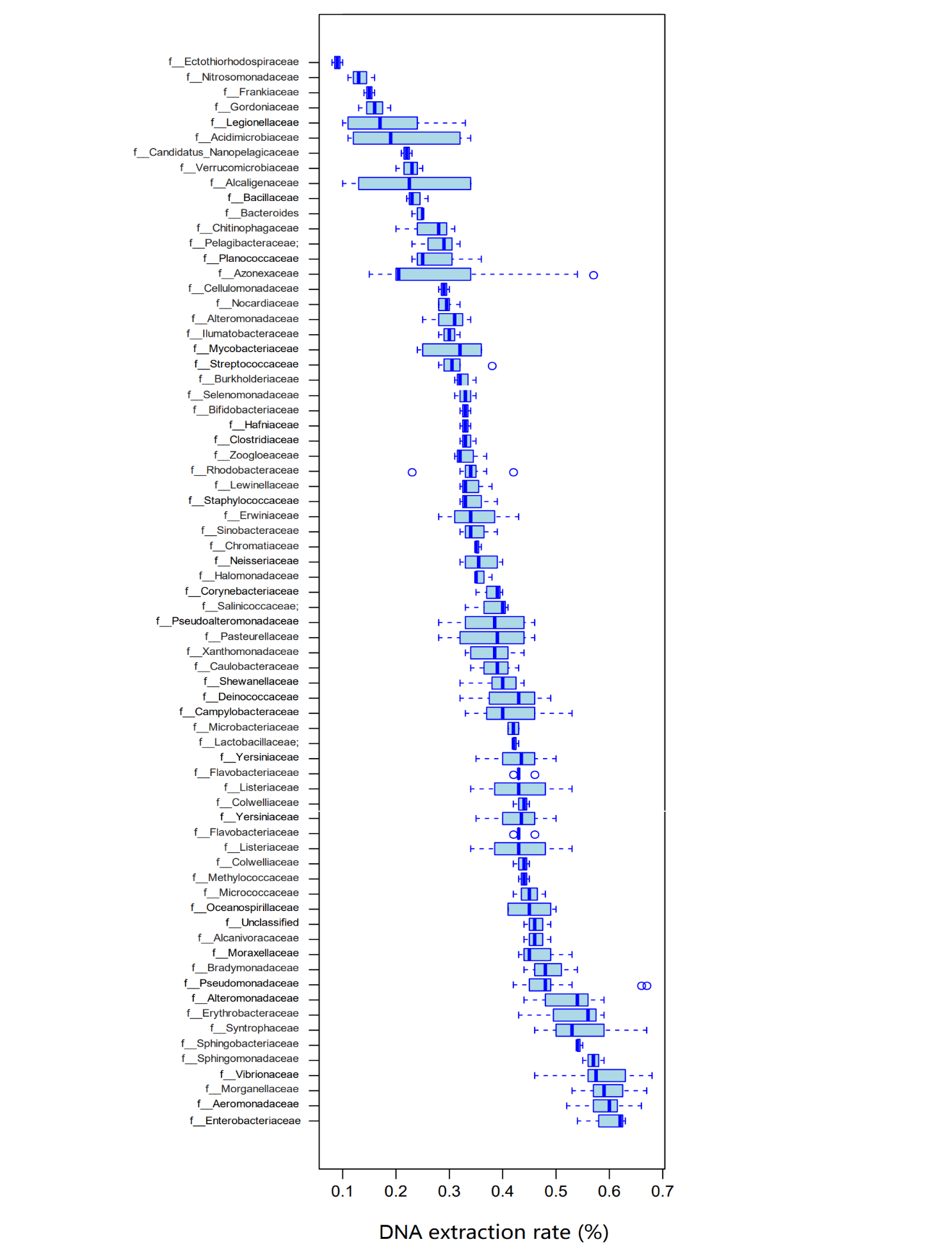


**Figure S4** Relative abundance estimates can misrepresent actual concentrations due to shifts in total bacterial load. Six examples, including *Acinetobacter baumannii*, *Acinetobacter pitti*, *Aeromonas caviae*, *Vibrio harveyi*, *Cronobacter sakazakii* and *Shewanella algae,* are displayed. Vertical bars show the relative abundance (%, left y-axis), solid lines indicate the inferred concentrations, and the dashed lines indicate absolute concentrations measured by qPCR. The dashed black line indicates the detection threshold for qPCR data. Arrows indicate time points when the relative abundance changes are discordant from the absolute concentration changes, which often occur when bacterial loads shift dramatically or when the relative abundance is low.


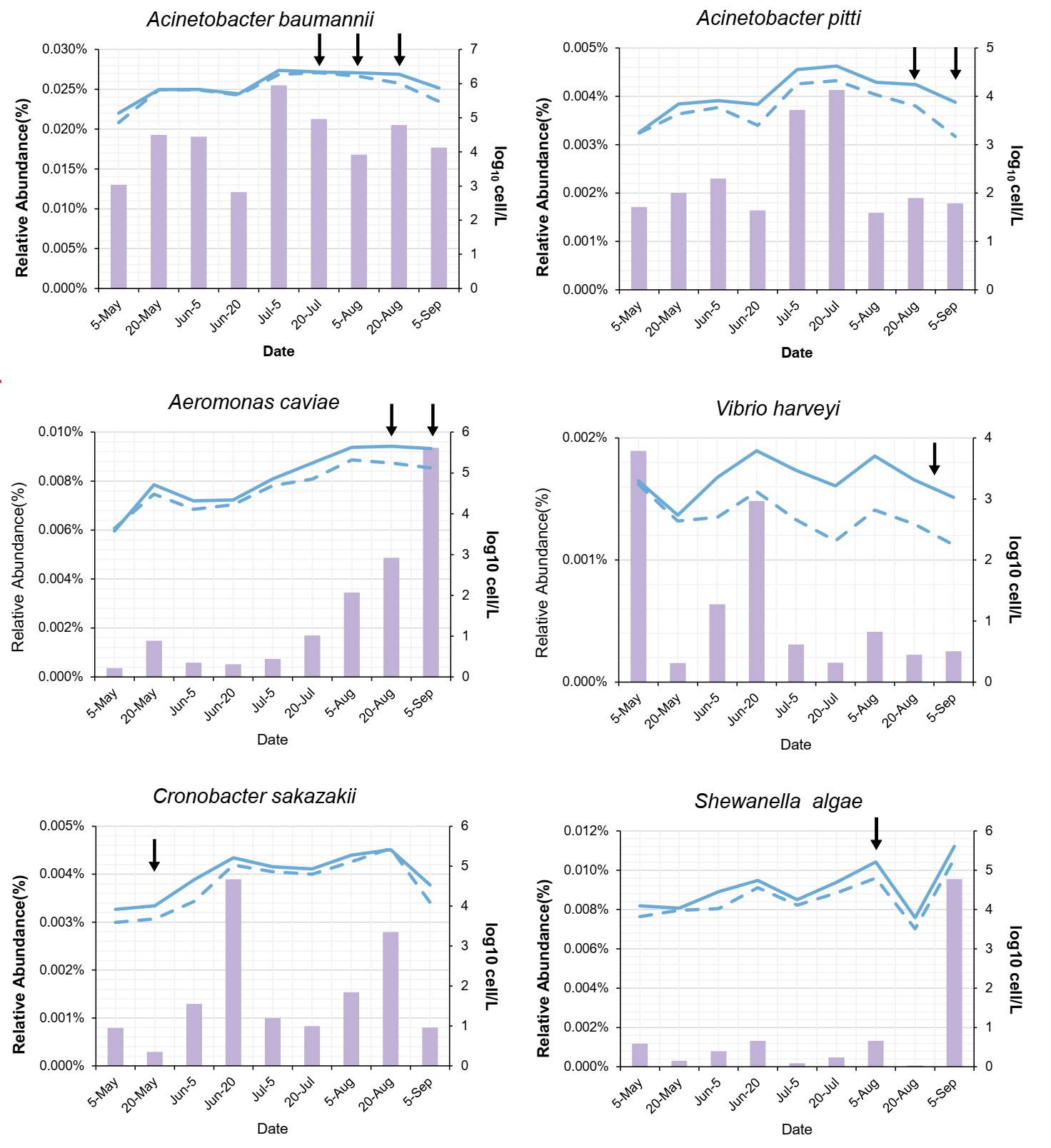


**Figure S5** Observed vs expected individual bacterial abundance of *Bacillus cereus* (A), *Salmonella enterica* (B), *Acinetobacter baumannii* (C), *Staphylococcus aureus* (D), *Vibrio parahaemolyticus* (E), and *Vibrio cholerae* (F). Expected individual bacterial abundance was measured by qPCR of individual species, while observed abundance was calculated using total bacterial load multiple with corrected relative abundance.


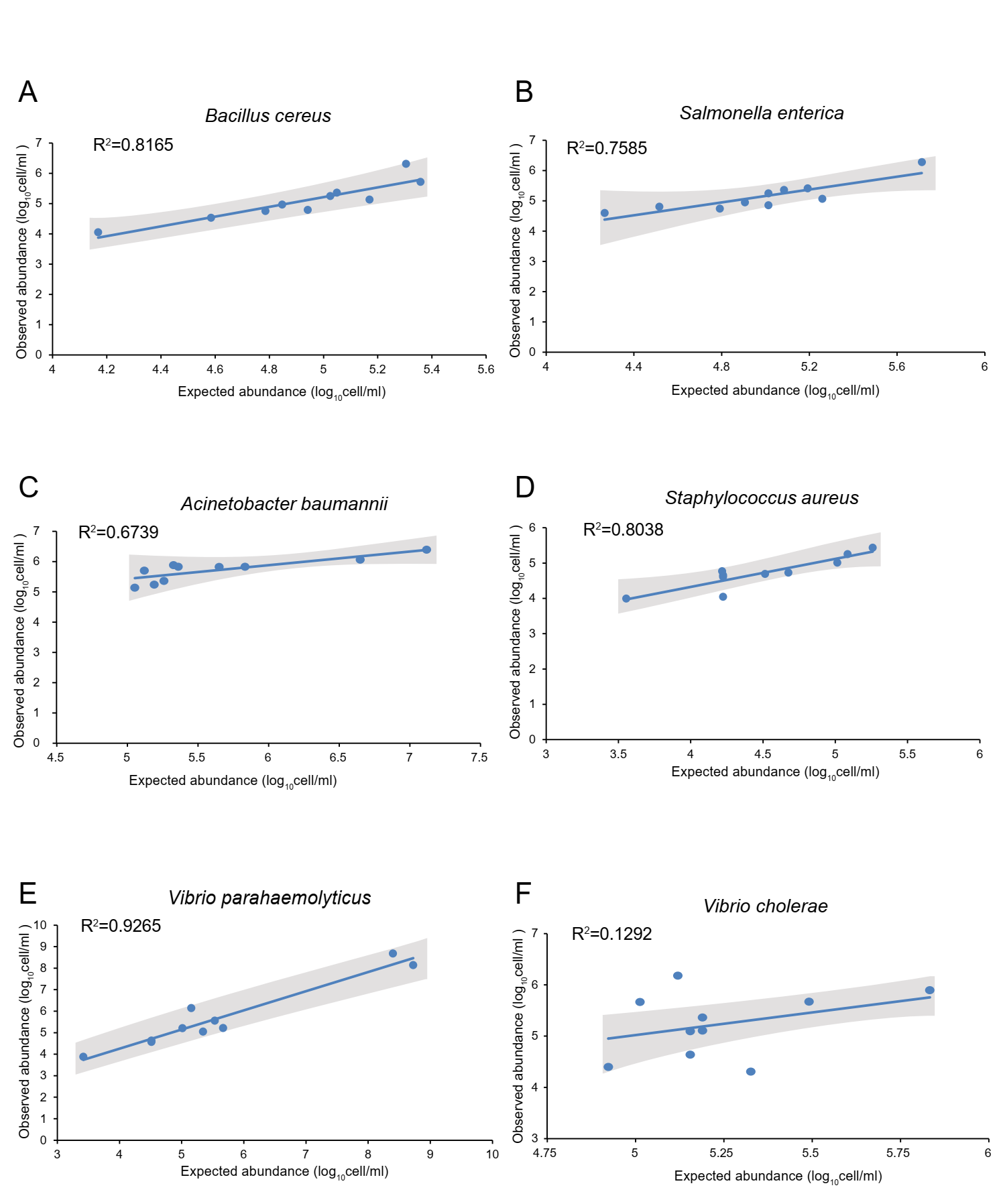


**Figure S6** Relative abundance (A) and N50 value (B) of MAGs obtained from metagenomic dataset in wastewater.

**
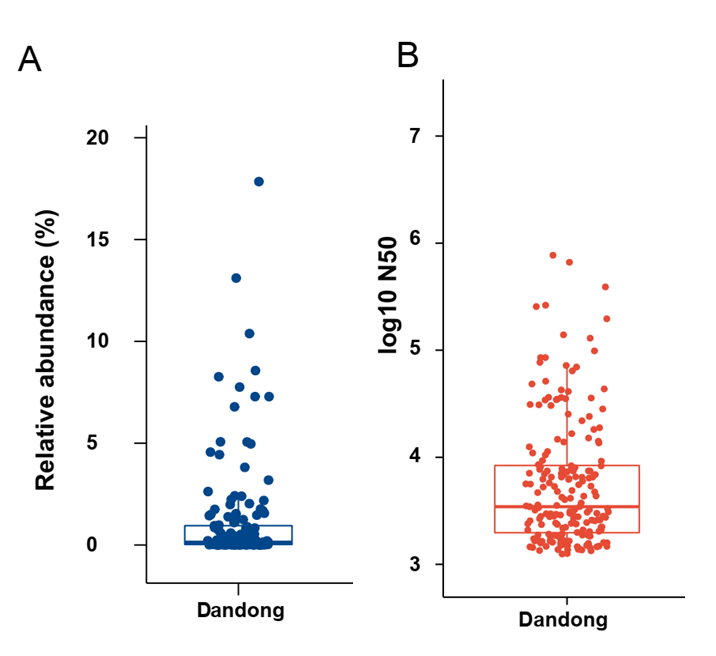
**
